# The correlation between promoter hypermethylation of VDR, CLDN, and CasR genes and recurrent stone formation

**DOI:** 10.1186/s12920-022-01265-1

**Published:** 2022-05-11

**Authors:** Fatemeh Khatami, Alireza Gorji, Mahdi Khoshchehreh, Rahil Mashhadi, Mahin Ahmadi Pishkuhi, Alireza Khajavi, Alireza Namazi Shabestari, Seyed Mohammad Kazem Aghamir

**Affiliations:** 1 Urology Research Center, Tehran University of Medical Sciences, Tehran, Iran; 2 Department of Pathology, University of California, Los Angeles, USA; 3 Pars Advanced and Minimally Invasive Medical Manners Research Center, Pars Hospital, Iran University of Medical Science, Tehran, Iran; 4 Faculty of Paramedical Sciences, Shahid Beheshti University of Medical Sciences, Tehran, Iran; 5 Department of Geriatric Medicine, School of Medicine, Tehran University of Medical Sciences, Tehran, Iran

**Keywords:** Promoter hypermethylation, Recurrent stone formation, Epigenetic

## Abstract

**Objectives:**

Recurrent Kidney stone formation is a main medical problem imposing a significant burden on both healthcare and the economy worldwide. Environmental and genetic factors have been linked to a bigger risk of kidney stone formation. We aim to assess the role of methylation on recurrent stone formation in three target genes.

**Methods:**

We aimed to check the association between promoter hypermethylation vitamin D receptor (VDR), calcium-sensing receptor (CaSR), and claudin 14 (CLDN14) genes in recurrent kidney stones. We enrolled 30 consecutive recurrent kidney stone formers (age 18–60 years) (cases) and 30 age and gender-matched controls.3. To identify promoter methylation, two target regions from each candidate gene were bisulfited after blood collection and DNA extraction. Methylation quantification was done through methylation-specific high resolution melting (MS-HRM).

**Results:**

The mean age of the patients and controls (mean ± SD) was 49.58 ± 14.23 years and BMI 36.12 ± 2.72. The methylation status in all six target regions was meaningfully different between the stone-former group and controls when methylation was considered in three clusters of unmethylated, methylated, and hypermethylated. A higher effect in VDR and CLDN was observed compare to CasR (*p*-value < 0.001, and < 0.005 versus *p*-value < 0.256).

**Conclusions:**

Methylation as an important epigenetic mechanism should be considered more in recurrent stone formations. Promoter hypermethylation of VRD and CLDN genes may have an essential role in recurrent kidney stones formations.

## Introduction

Genetic and epigenetic changes are two main elements that can increase the risk of kidney stones formation. Contrary to genetic change in which the DNA sequence changed, the gene expression pattern changed reversibly with no change in the DNA nucleotide sequences in epigenetic modifications like DNA methylation. Recurrent kidney stone formation is a multifactorial disease that can be the consequence of several environmental and lifestyle factors. Most genes involved in the condition are essential for transporting materials in and out of cells. Over the last decade, several epidemiological studies have shown high kidney stone disease in all age groups [1, 2]. Several risk factors are studied for recurrent urolithiasis like caffeine intake, dietary intake, smoking, alcohol, physical activity [3, 4] Epigenetic modifications like DNA methylation in CpG islands define spatial conformation of chromatin to regulate gene expression. DNA methylation change can be the consequence of environmental factors.

Some genes are the most highlighted ones in recurrent kidney stones, like Vitamin D Receptors (VitD R), Claudin 14 (CLDN14), and Calcium-sensing receptor (CaSR) [5, 6]. Despite the established importance of these three genes in kidney stone formation, their promoter methylations have not evaluated yet. In this study, for the first time we aimed to determine the promoter methylation status of VitD R, Claudin, and CaSR in recurrent kidney stone formation.

## Materials and methods

### Study population and specimen's collection

The study was run under the Tehran University of Medical Sciences ethical committee (IR.TUMS.SINA HOSPITAL.REC.1399.033) after receiving the written informed consent from both patients and controls. All the methods were in accordance with the relevant institutional or in accordance with the declaration of Helsinki. A total number of sixty patients were recruited, of which thirty were recurrent stones patients, and the other thirty were individuals with no kidney stones nor a positive history of urolithiasis. A recurrent stone former was defined as an individual with at least two symptomatic episodes in less than six months intervals. Patients were aged between 18 and 90 years old with no systemic disorders. Patients with a history of known metabolic, gastrointestinal, hepatic, renal, or endocrinological diseases were excluded. A blood sample was sent for serum urea, creatinine, calcium, phosphate, magnesium, and uric acid concentrations, as well as vitamin D3, calcitonin, parathyroid hormone (PTH), and alkaline phosphatase levels.

### DNA extraction and bisulfite treatment

A total amount of 4–6 ml blood was collected in EDTA tubes. Then genomic DNA was extracted from the patients with recurrent kidney stones and their age and gender-matched control counterparts by a DNeasy Blood & Tissue Kit (Qiagen, Hilden, Germany) based on the manufacturer's instruction. Then genomic DNA was treated for Bisulfite modification of DNA by the "EZ DNA Methylation‐Gold™ Kit" (Cat No: D5005, Zymo Research) according to the manufacturerʼs protocol. This step is essential for discrimination between methylated CpG island and unmethylated ones because bisulfite Conversion is a procedure in which DNA (gDNA) is denatured (made single-stranded) and after sodium bisulfite treatment, delamination of unmethylated Cytosines into Uracil's happens, whereas methylated cytosines (both 5-methylcytosine and 5-hydroxymethylcytosine) stay unaffected.

### Methylation analysis by MS-HRM

**Fig. 1:**
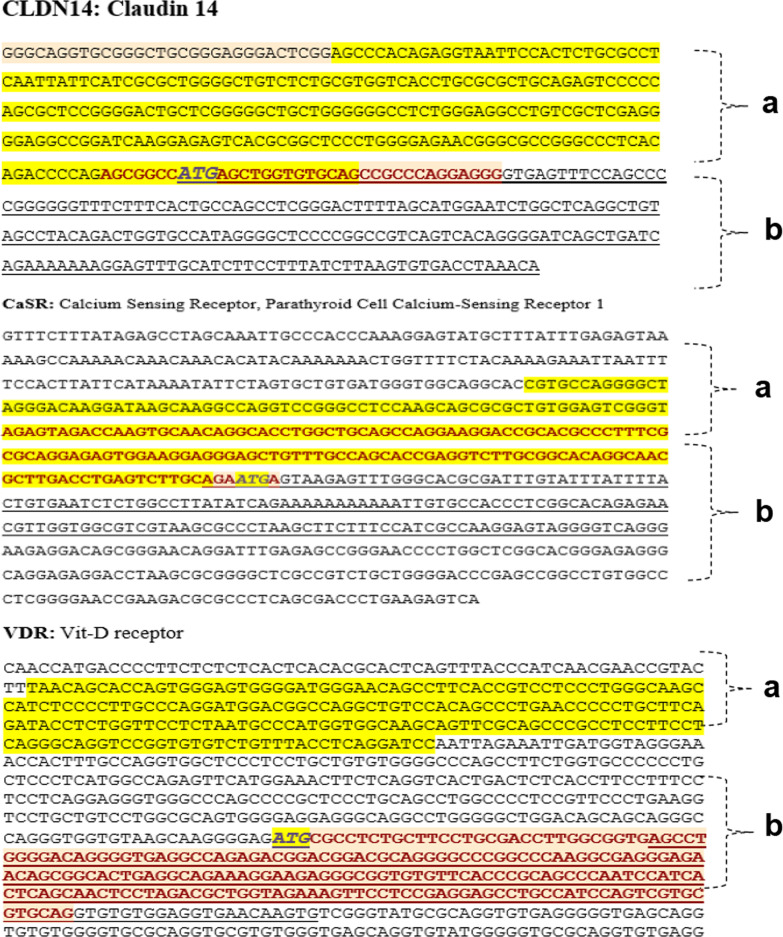
The sequence of six targeted promoter regions of Vitamin D Receptors (VitD R), Claudin 14 (CLDN14), and Calcium-sensing receptor (CaSR) genes for MS‐HRM analysis

For detecting the quantity of CpG methylated islands, the Methylation Specific High-Resolution Melting (MS‐HRM) was applied. Specific primers were designed to evaluate the promoter methylation of three target genes VitD R, Claudin, and CaSR (Table 1). The primer sets for all MS-HRM assays were prepared according to the principles recently set out to compensate for PCR bias [7]. These primers were used to amplify six target promoter regions indicated by "a" and "b ". The locus of the primers and target amplicons are presented in Fig. 1. The MS‐HRM analyses consisted of the following three main steps: holding step (95 degrees Celsius for 15 min), trailed by 38 cycles of 95 degrees Celsius for 20 s; annealing temperature (ranging from 47 degrees Celsius to 60 degrees Celsius) for 30 s; and extension time of 72 degrees Celsius for 30 s leading to the last step of the Melting curve. The melting curve includes heating up to 94 degrees Celsius for 15 s and 60 degrees Celsius for 45 s leading to 65 degrees Celsius for 20 s, then heating up uninterruptedly to 94 degrees Celsius with data recording for every 0.5 degrees Celsius temperature rise. All reaction combinations contained 10 µl of master mix (Cat No: A325406; Amplicon), 20 pmol forward and reverse primers, and 2 µl (almost 20 ng) of bisulfite modified DNA template in the closing volume of 20 µl. MS‐HRM experiments were performed by ABI Step One Plus System.

**Table 1:** The sequence of specific primers for studying six candidate promoter regions of VitD R, Claudin 2, CaSR for high‐resolution melting analysis

Gene	Locus	Primers (Forward and Reverse)	Length of Amplicon	Tann (degrees Celsius)	Number of covered CpG dinucleotides within the amplicon
CLDN14	a	F: AGTTTATAGAGGTAATTTTATTTTG R: CTACACACCAACTCATAACC	272 bp	50	18 CpG sites
CLDN14	b	F: GGTTATGAGTTGGTGTGTAGT R: TATTTAAATCACACTTAAAAT	214 bp	52	5 CpG Sites
CaSR	a	F: GTGTTAGGGGTTAGGGATAAGGATA R: TCATTCTACAAAACTCAAATCAAAC	218 bp	59	11 CpG Sites
CaSR	b	F: AGAATGAGTAAGAGTTTGGGTA R: CTCTTCCCTAACCCCTACTCCT	175 bp	55	8 CpG Sites
VitD R	a	F: TAATAGTATTAGTGGGAGTGGGGAT R: AAATCCTAAAATAAACAAACACACC	215	56	5 CpG Sites
VitD R	b	F: AGTTTGGGGATAGGGGTGAGGTTA R: CACTTATTCACCTCCACACACCTAC	168	55	11 CpG Sites

### Statistical analysis

The methylation status in control and stone-former groups was compared using the chi-squared test. The odds ratio (OR) of methylation status in the case group was calculated for each promoter region of three targeted genes, VitD R, Claudin, and Cas R and were compared to the control. The description for outside normal serum calcium was also estimated for two groups and was expressed as mean and standard deviation (SD). A statistically significant *p*-value of less than 0.05 was considered. Statistical Package did all analyses for Science Software (SPSS, version 17.0; SPSS Inc., Chicago, IL).

## Results

A total of 30 stone-forming patients and 30 age and sex-matched counterpart controls were recruited in the study. The mean age of the patients and controls (mean ± SD) was 49.58 ± 14.23 years and BMI 36.12 ± 2.72 (Table 2).

**Table 2:** Demographic and clinical information of recurrent stone formers and controls

Variable		Recurrent stone former cases (*N* = 30)	Control (*N* = 30)	*P*-value
Gender (Female)		9 (30.0%)	10 (33.3%)	0.781
Hospitalization under six hours		0 (0.0%)	2 (6.7%)	0.492
Marital Status	Single	11 (39.3%)	13 (46.4%)	0.589
	Married	17 (60.7%)	15 (53.6%)	
UTI		12 (40.0%)	5(16.6%)	0.052
Heart counseling		15 (50.0%)	16 (53.3%)	0.796
Blood creatinine (Abnormal)		5 (18.5%)	0 (0.0%)	0.051
Blood sugar		5 (16.7%)	6 (20.0%)	0.739

The findings of Table 2 show the patient's demographic and characteristics in the case group compared to the control group. Only the Creatinine variable was marginally significantly different between the two groups (*p*-value = 0. 051). The frequency of other variables did not show a significant difference between case and control groups. MS-HRM methylation was defined as the unmethylated (methylation < 9%), methylated (9–29%) and highly/ hypermethylated (> 29%) [8].

**Fig. 2:**
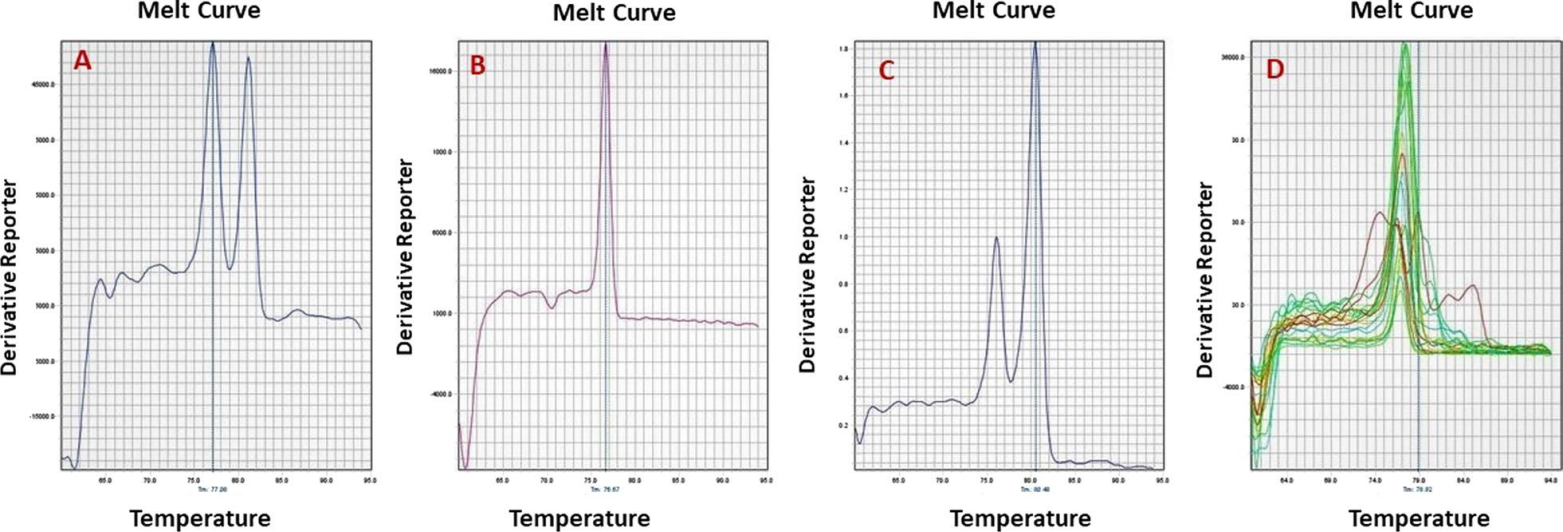
The resulting graph of MS-HRM for 50%, 100%, 75% methylated DNAs. **A** Indicated to 50% methylation because half of amplicon are melted (double stranded DNA converts to single stranded DNA) at lower temperature which indicated to unmethylated cytosine replacement by uracil. **B** indicated to 100% unmethylated because all DNAs are melted at lower temperature. **C** Represent the 75% methylated promoter because ¼ of DNA are melted at lower temperature and ¾ are melted at higher temperature. **D** showed all runs over each other which should consider one by one like **A**, **B**, and **C**

MS-HRM was analyzed based on comparing melt profiles of experimental samples to profiles from DNA with known methylation levels. Universally (or 100%) methylated DNA is commercially available. For a source of unmethylated DNA, scientists often isolate DNA from blood mononuclear cells. For this study, the Sigma-Aldrich CpGenome™ Human Methylated & Non-Methylated DNA Standard Set (Cat# S8001) were used as the 0% and 100% methylated DNA. 100% methylated and 0% methylated DNA of equal concentration were then mixed in different ratios to mimic DNA samples with defined DNA methylation levels. The methylation quantification of each test sample was evaluated in triplets (Fig. 2).

The methylation status in all six target regions was statistically significantly different between the stone-former group and controls when methylation was considered in three clusters of unmethylated, methylated, and hypermethylated (Table 3). As we shown in Fig. 1, the VDRa is the distant promoter, and VDRb is the proximal one to the translation initiation codon (ATG). For CLDNa and CLDNb is the same as the CasRa and CasRb, in which "a" means the target sequence is distal and "b" is the proximal ones to the translation initiation codon (ATG).

**Table 3:** The methylation status of six target promoter regions in VDR, CasR, CLDN14 when methylation defined in three subgroups of the unmethylated (methylation < 9%), methylated (9–29%), and highly/hypermethylated (> 29%)

Gene		Recurrent stone formers (*N* = 30)	Control (*N* = 30)	*P*-value
VDRa	Unmethylated	2 (6.7%)	12(42.9%)	0.006*
	Methylated	16(53.3%)	9(32.1%)	
	Hypermethylated	12(40.0%)	7(25.0%)	
VDRb	Unmethylated	1(3.4%)	10(34.5%)	0.007*
	Methylated	14(48.3%)	12(41.4%)	
	Hypermethylated	14(48.3%)	7(24.1%)	
CLDNa	Unmethylated	4(14.8%)	15(50.0%)	.004*
	Hypermethylated	10(37.0%)	11(36.7%)	
	Hypermethylated	13(48.1%)	4(13.3%)	
CLDNb	Unmethylated	4(14.8%)	13(43.3%)	< 0.001*
	Methylated	6(22.2%)	13(43.3%)	
	Hypermethylated	17(63.0%)	4(13.3%)	
CasRa	unmethylated	2(7.1%)	6(20.0%)	< 0.001**
	Methylated	11(39.3%)	23(76.7%)	
	Hypermethylated	15(53.6%)	1(3.3%)	
CasRb	Unmethylated	1(3.4%)	6(20.0%)	< 0.001**
	Methylated	12(41.4%)	21(70.0%)	
	Hypermethylated	16(55.2%)	3(10.0%)	

^*^Less than 0.005

^**^less than 0.001

**Fig. 3:**
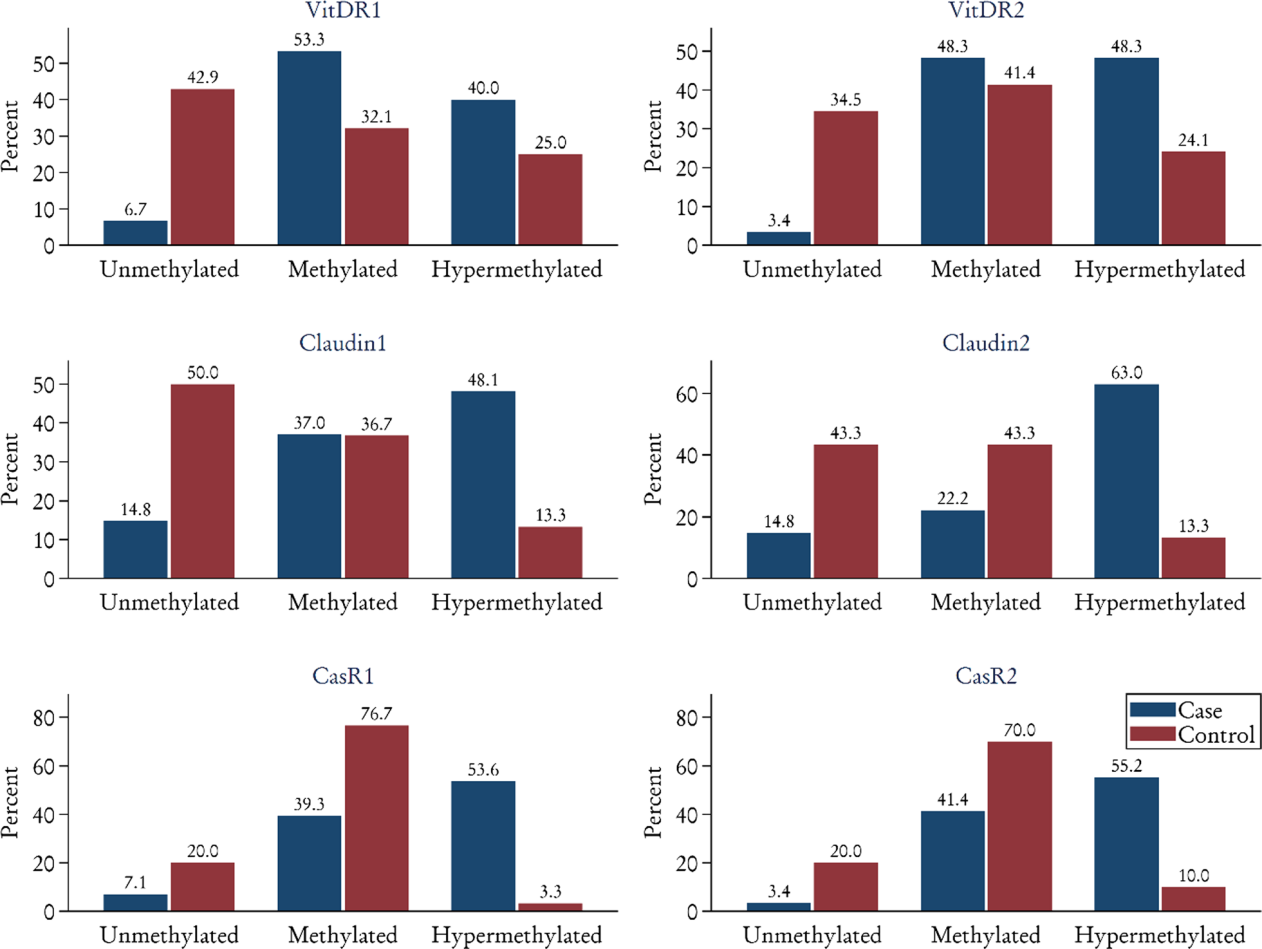
The difference of methylation status of six target promoter regions between cases and controls

When we consider the methylation status as methylated (methylation < 9%) and unmethylated (methylation ≥ 9%), the methylation CLDNb, CasRa, and CasRb did not show the significant difference between the two groups of stone-former cases and controls (Table 4). Compared to the healthy controls, the VDR was the only gene in which hypermethylated promoter was significantly increased in the recurrent stone-former group. In the CLDN gene, the distal promoter region was statistically significantly more hypermethylated in recurrent stone-formers (Table 5). The promoter methylation of the Receptor of Vit D is the most critical gene in recurrent stone formation (Fig. 3).

**Table 4:** The methylation status of six target promoter regions in VDR, CasR, CLDN14 when methylation is defined in three subgroups of the Unmethylated (methylation < 9%), and Methylated (methylation ≥ 9%)

Gene		Recurrent stone formers (*N* = 30)	Control (*N* = 30)	*p*-value
VDRa	Unmethylated	2 (6.7%)	12(42.9%)	0.001*
	Methylated	28(93.3%)	16(57.1%)	
VDRb	Unmethylated	1(3.4%)	10(34.5%)	0.005*
	Methylated	28(96.6%)	19(65.5%)	
CLDNa	Unmethylated	4(14.8%)	15(50.0%)	0.005*
	Hypermethylated	23(85.2%)	15(50.0%)	
CLDNb	Unmethylated	4(14.8%)	13(43.3%)	< 0.019*
	Methylated	23(85.2%)	17(56.7%)	
CasRa	unmethylated	2(7.1%)	6(20.0%)	< 0.256**
	Methylated	26(92.9%)	24(80.0%)	
CasRb	Unmethylated	1(3.4%)	6(20.0%)	< 0.150**
	Methylated	26(92.9%)	24(80.0%)	

The methylation status was not associated with the age and gender of recurrent stone formers (Table 5)

**Table 5:** Hypermethylated promoter in recurrent stone formers to cmpare gender and age

Gene	Female	Male	*P*-value
VDRa	9 (100%)	19 (90.5%)	0.483
VDRb	9 (100%)	19 (95.0%)	0.690
CLDNa	9 (100%)	14 (77.8%)	0.268
CLDNb	9 (100%)	14 (77.8%)	0.268
CasRa	8 (88.9%)	18 (94.7%)	0.575
CasRb	8 (88.9%)	18 (94.7%)	0.575

	Age < 60	Age ≥ 60	*P*-value
VDRa	22 (91.7%)	6 (100%)	0.634
VDRb	22 (95.6%)	6 (100%)	0.793
CLDNa	18 (81.8%)	5 (100%)	0.561
CLDNb	18 (81.8%)	5 (100%)	0.561
CasRa	20 (90.9%)	6 (100%)	0.611
CasRb	20 (90.9%)	6 (100%)	0.611

In 30 recurrent stone formers, 21 were male (70%) and 24 were under 60 (80%). Hypermethylated for each target locus are presented

The methylation status of six target promoter regions was correlated with each other. The correlation coefficients for this correlation were estimated by the kappa agreement test and shown in Table 6. The most correlated ones between the two target intragenic regions were CasRa and CasRb, then VDRa and VDRb, and between two different genes of VDR with CLDN.

**Table 6:** Correlation analysis of methylation status between six target promoter regions evaluated by kappa agreement test

	VDRa	VDRb	CLDNa	CLDNb	CasRa	CasRb
VDRa	1	0.895	0.7	0.687	0.397	0.397
VDRb	0.895	1	0.587	0.66	0.473	0.473
CLDNa	0.7	0.587	1	0.837	0.452	0.452
CLDNb	0.687	0.66	0.837	1	0.48	0.41
CasRa	0.397	0.473	0.452	0.41	1	1
CasRb	0.397	0.473	0.452	0.41	1	1

## Discussion

Methylation as an important epigenetic mechanism should be considered more in recurrent stone formations. Promoter hypermethylation of VRD and CLDN genes may have an essential role in recurrent kidney stones formations. We select vitamin D receptor (VDR), calcium-sensing receptor (CaSR), and claudin 14 (CLDN14) genes based on literature and meta-analysis. Only these three genes were studied in this work. This deficiency needs to be reflected. We select the genes that their gene expression regulation is controlled by promoter methylation. Because there is not previous study on methylation and just SNPs are discussed in Urolithiasis our study can be the good starting point for such studies. Renal stone formation risk factors have classically reflected environmental, geographic, and dietary issues, often based on population studies. Several treatment strategies are considered for the stone treatment depending on the stone size and skin-to-stone distance [9–13]. Moreover, the heritability of renal stones has also been proven. In twin studies, the estimated heritability of kidney stones was reported at 56%, and this has been supported by additional studies measuring the familial pattern of stones [14]. More recently, there has been a comprehensive exploration for rare monogenetic bases of renal stones by means of selected stone-forming populations [15–17].

Hypercalciuria is integral in the pathogenesis of stone formation that can be arise through calcium deposition in the renal papilla. The etiology of hypercalciuria is not well understood but includes augmented bone resorption, calcium hyperabsorption in the intestine, and lessen renal reabsorption. Calcium reabsorption happens mainly in the proximal tubule that about sixty percent of calcium filtered through the glomerulus is reabsorbed. The calcium carriage is done by claudins, an important components of the epithelial tight junctions found at tight junctions. Tight junctions are a surrounding cellular blockade that controls the flow of molecules in the intercellular space between the cells of an epithelium. The claudins perform selective transport and control the movement of solutes through the epithelium.

Some infrequent monogenic forms of stone formation can originally look a fine-print issue; lost detects of monogenetic reasons of nephrolithiasis might consequence to sub-optimal action, complications, and disappointment to screen at-risk family members [18–20]. Screening common pathogenic mutations by some novel high-throughput genomics-based strategies to assay GWAS SNPs, Sequencing by synthesis technology, Bead Array microarray technology, and is rapidly becoming an available diagnostic tool [15, 21]. In studies of hypercalciuric and hypocalciuric patients' monogenic origins and infrequent alleles were not clearly recognized [22]. CLCN5 variants were a reasonable candidate for idiopathic hypercalciuria. However, those variants were found to be rare [23].

Several studies analyzed the associations of VDR gene expression with urolithiasis risk in different ethnic groups [24]. A meta-analysis by Imani D et al. indicated that although their study did not emphasize the relationship of FokI, TaqI, BsmI, and ApaI in the general sampled examination, but it proposes that ApaI and TaqI SNPs are linked to the bigger risk of urolithiasis in East-Asian and Caucasians populations [5]. It was indicated that urinary calcium excretion has increased in response to vitamin D (VitD) supplements, at least in some groups of kidney stone formers. It has been proposed that predisposed individuals may develop hypercalciuria and kidney stones in response to vitamin D supplements [25]. The VDR FokI polymorphism may be a good candidate for calcium oxalate stone disease marker. The epidemic of Randall plaque-associated renal stones in young patients can be the implication of altered vitamin D response [26].

Regarding CLDN, our data suggested the lower gene expression of CLDN can be the result of the promoter hypermethylation of CLDN. Similarly, Curryn JN and colleagues also described that a family with a rare missense variant in the CLDN gene has noticeable hypercalciuria, and kidney stone disease findings may indicate that CLDN can be a crucial regulator of calcium excretion and a potential target for therapies to prevent kidney stones [27]. Also, it was shown that single nucleotide polymorphisms of CLDN are significant in hypercalciuria and kidney stone formation [28]. The CLDN expression is downregulated in many pathologies, like cancer, inflammation, and fibrosis. The CLDN play an important role in energy-efficient ion and water transport in the proximal tubules of the kidneys and in the intestines. Notably, substantial studies highlighted a critical role for this protein as a modulator of critical cellular pathways related to stone diseases. Cell signaling pathways and molecular mechanism that are over activated in different conditions can change the claudin-2 expression, and a respectable association occurs concerning disease stage and claudin-2 overexpression. Additional, loss- and gain-of-function research presented that direct alterations in claudin-2 expression influence critical metabolic pathways in human cells. These properties seem to be intermediated by changes in critical signaling pathways [29].

The CaSR gene expression change commonly highlighted in the parathyroid glands and in renal tubules that control Parathyroid hormone (PTH) secretion. The kidneys are the main route for the excretion of salt and water and have an important role in the control of body fluid osmolality and Intracellular fluid volume (ICFV) and extracellular fluid volume (ECFV). The kidney controls electrolyte and water excretion, fix different tubular segments' functions. In particular, CaSR decreases both passive and active calcium reabsorption in distal tubules, raises phosphate reabsorption in proximal tubules, and triggers proton and water excretion in collecting ducts. Therefore, it can be an important gene for causing calcium nephrolithiasis. Our data indicated a less critical role of Cas R epigenetic control in recurrent kidney stones formation. Some contradictory results showed an essential function of Cas R [30]. One possible explanation might be that Cas R cis's gene expression is not regulated by epigenetic mechanisms like methylation.

Finally, we can say promoter hypermethylation of VRD and CLDN genes has an essential role in recurrent kidney stones formations.

## Data Availability

All datasets used and/or analyzed during the current study are provided and has no additional data.
